# Gelation Behaviour of Pluronic F127/Polysaccharide Systems Revealed via Thioflavin T Fluorescence

**DOI:** 10.3390/gels9120939

**Published:** 2023-11-30

**Authors:** George-Alin Balan, Aurica Precupas, Iulia Matei

**Affiliations:** “Ilie Murgulescu” Institute of Physical Chemistry of the Romanian Academy, 202 Splaiul Independentei, 060021 Bucharest, Romania

**Keywords:** thioflavin T, Pluronic F127, alginate, hyaluronic acid, steady-state fluorescence, synchronous fluorescence

## Abstract

Fast, reliable methods for characterizing the micelle-to-gel transition in emerging Pluronic F127/polysaccharide materials are essential for tailoring their applications as in situ gelling delivery systems. This study describes a simple fluorimetric method based on the response to gelation of the molecular probe thioflavin T (ThT). The techniques employed are (second derivative) steady-state and synchronous fluorescence. The capabilities of ThT as gelation reporter are tested for three model systems: Pluronic F127 (P16.6%), Pluronic F127/alginate (P16.6%ALG2%) and Pluronic F127/hyaluronic acid (P16.6%HA0.5%). We demonstrate that the changes in the short and long wavelength emissions of ThT allow accurate determination of the critical gelation temperatures in the investigated systems. The spectroscopic data providing information at molecular level are complemented with differential scanning microcalorimetric results revealing additional macroscopic insight into the micellization process. The gelation study is preceded by a solvatochromic analysis of ThT.

## 1. Introduction

**Scheme 1:**
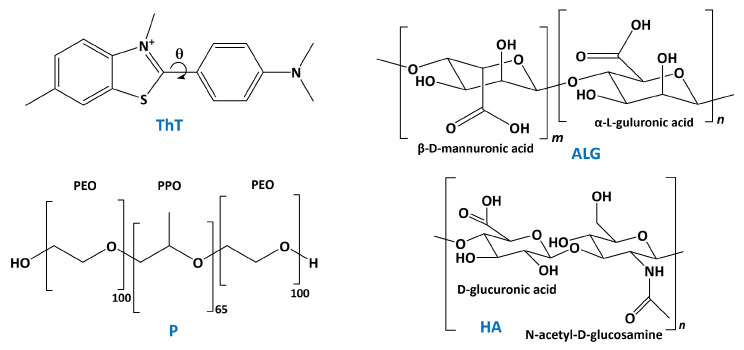
Chemical structures of thioflavin T (ThT), Pluronic F127 (P), alginic acid (ALG) and hyaluronic acid (HA); PEO = poly(ethylene oxide) block and PPO = poly(propylene oxide) block.

The molecular probe Thioflavin T (ThT) has been extensively investigated regarding its interaction with macromolecular hosts like cyclodextrins [1] and cucurbiturils [2], with ionic and non-ionic micellar systems [3,4], and with biological targets like polypeptides/proteins [5,6] and DNA [7]. This is due to the extraordinary sensitivity of its spectral signatures to microenvironmental properties (polarity and viscosity) that originates in its behaviour as a molecular rotor [8]. Briefly, ThT consists of a benzothiazole ring and a dimethylaniline ring connected through a σ(C–C) bond allowing intramolecular rotation (dihedral angle θ, Scheme 1). Other key structural features are the positive charge in the benzothiazole ring (at pH < 10) and the tertiary dimethylamino group in the benzyl ring. Since charge transfer occurs from the HOMO molecular orbital localized on the dimethylaniline ring to the LUMO localized on benzothiazole [9], it is evident that the value of θ largely determines the spectral properties of ThT. Its photophysical behaviour in nonviscous media is governed by the stabilization of either the emissive locally excited (LE) state (θ ~ 37 deg) or the weakly emissive twisted intramolecular charge transfer (TICT) excited state (θ ~ 90 deg). In viscous media, or by binding to a substrate in a “locked” conformation, the nonradiative (torsional relaxation) deactivation channel of ThT is suppressed, leading to fluorescence enhancement [10,11].

Despite early interest in its photophysics, presently ThT is employed mostly as staining dye in amyloid assays [12], aiming for early diagnosis and disease-specific therapy of amyloidosis. Applications of ThT to gel systems are scarce [13,14], but a recent study by Mata et al. [15] has shown that the induced circular dichroism signal of ThT can be used to evidence the formation of a self-assembled hydrogel between a peptide amphiphile and a sugar-based low-molecular-weight gelator (dibenzylidene-D-sorbitol). These results prompted us to conduct an investigation on the fluorescence response of ThT during the gelation of some Pluronic F127/polysaccharide (alginate—ALG and hyaluronic acid—HA) model systems. The chemical structures of ThT along with those of the system constituents are depicted in Scheme 1.

The selection of these gelling systems has been made considering the previous work conducted in our laboratory [16,17,18,19] that provides us with the essential reference information on temperature-induced micellization and gelation processes in Pluronic F127 alone and in the presence of a modulator of its encapsulation properties (2-hydroxypropyl-β-cyclodextrin). Alginate (ALG) and hyaluronic acid (HA), the two polysaccharides selected for this study, are negatively charged at the working pH and can thus promote interaction with oppositely charged ThT. Such Pluronic/polysaccharide systems are becoming key components in today’s pharmaceuticals due to their application as in situ gel-forming delivery vehicles [20,21,22]. Data in the literature indicate that polysaccharide addition represents a convenient means of ensuring the stability of the gel against dissolution and of increasing the gel strength and self-healing ability [23,24].

The difference in hydration between PEO blocks (more hydrophilic) and PPO blocks (more hydrophobic) lies at the core of the temperature-sensitive processes occurring in Pluronic F127 systems [25]. As the temperature increases, progressive dehydration of PPO chains leads to the formation of the micellar core at the critical micellization temperature (cmt). Further temperature increase determines the progressive dehydration of PEO chains from the micelle corona, causing chain entanglement between adjacent micelles and culminating in the micelle-to-gel transition occurring at the critical gelation temperature (cgt). This phase transition is influenced by the presence of other system constituents that reside at the interface between Pluronic F127 micelles and water [26]. They may cause significant microstructural changes in the sample that we believe ThT to be responsive to.

To our knowledge, this is the first investigation exploring the potential of ThT as a fluorescent reporter of gelation. Our study also aims to gather insight into the nanoscopic changes in polarity/viscosity associated with the molecular reorganization processes accompanying the micelle-to-gel transition in Pluronic/polysaccharide systems. Two fluorescence methods, steady-state and synchronous, are investigated in order to assess their capabilities, and second-derivative synchronous fluorescence is used to reveal additional spectral features related to the micellization process. The latter process is also followed by a complementary method, differential scanning microcalorimetry (μDSC). Our results indicate that the fluorescence response of ThT represents a suitable method for accurately determining the temperature corresponding to the transition from micelle to gel, that is, the critical gelation temperature (cgt), in Pluronic/polysaccharide systems.

## 2. Results and Discussion

### 2.1. Solvatochromic Study of ThT

The complexity of ThT photophysics, along with the observation that some anionic compounds may promote the electrostatic self-assembly of ThT into dimers/higher aggregates, leading to large bathochromic shifts in the fluorescence spectra [27,28], determined us to begin our investigation with a preliminary solvatochromic study. As such, the emission and excitation spectra of ThT at concentrations in the range 10^−6^–10^−4^ M have been recorded in three solvents varying in polarity and viscosity: methanol (ε = 32.70, η = 0.5 cP at 25 °C), diethylene glycol (ε = 31.70, η = 35.7 cP) and glycerol (ε = 46.5, η = 934 cP) [29]. This is necessary in order to select the optimal ThT concentration to be used in the gelation studies, and the λ_ex_ and Δλ parameters to be used in the steady-state and synchronous experiments (vide infra). This study also provides insight into the degree to which the polarity and viscosity of the microenvironment hinder torsional relaxation and stabilize the emissive species. We note that the highly viscous glycerol is a good indicator of ThT photophysics in Pluronic F127 solutions.

Selected steady-state fluorescence and excitation spectra of ThT in glycerol, at different ThT concentrations, are shown in Figures S1 and S2 in the Supplementary Material, respectively. At the lowest concentration, 10^−6^ M, two main emissions are observed: a short wavelength fluorescence band with a maximum at λ_f_ = 420 nm (λ_ex_ = 360 nm) and a long wavelength fluorescence band with two maxima at λ_f_ = 480 and 520 nm (λ_ex_ = 420 nm). The atypical short wavelength emission arises from a subset of ThT conformers with a broken π-electron conjugated system, due to a low energy barrier between the conformations with θ = 37 deg (ground state energy minimum) and θ = 90 deg [30,31]. For the conformers twisted at ~90 deg, the dimethylaniline and benzothiazole rings behave as independent chromophores, leading to hypsochromically shifted excitation and emission spectra.

The position of the long wavelength emission does not vary significantly with ThT concentration or solvent polarity. At λ_ex_ = 420 nm, which corresponds to the absorption maximum, λ_a_, of ThT in Pluronic F127 (vide infra), the long wavelength fluorescence is maximal at a ThT concentration of 10^−5^ M, followed by self-quenching in more concentrated solutions. For this reason, the 10^−5^ M concentration was selected to be used in the gelation studies. The emissions at 480 and 520 nm are favoured to the same extent by the increase in ThT concentration (F_480 nm_/F_520 nm_ ~ 1.5 at [ThT] = 10^−6^ M and 10^−4^ M) on the expense of the short wavelength emission. The presence of dimers was not observed at the ThT concentrations employed.

The broad feature of the two ThT emissions is due to the presence of a wide distribution of conformers [10,31] that is determined by the ThT concentration and by the polarity and viscosity of the microenvironment. The latter dependence becomes evident when comparing the spectra of ThT in glycerol (Figure S1) to those in diethylene glycol and methanol (Figures S3 and S4). At [ThT] = 10^−6^ M in diethylene glycol, an intermediary solvent in terms of viscosity between glycerol and methanol, the spectral signature of ThT is similar to that in glycerol regarding the relative intensities of the short and long wavelength emissions. Nevertheless, at this concentration, the short wavelength emission is observed at 440 nm as opposed to 420 nm in glycerol. Starting from [ThT] = 5 × 10^−5^ M, the long wavelength emission becomes dominant. In methanol, which has similar polarity to diethylene glycol but is nonviscous, the spectral signature of ThT is strikingly different, with an extremely weak long wavelength emission, irrespective of ThT concentration. In this solvent, intramolecular rotation occurs freely, and the fluorescence properties solely depend on the stabilization of the TICT (θ ~ 90 deg) state by the solvent.

Since the width of the steady-state fluorescence bands is directly related to the population of conformers, we decided to inspect the features of the synchronous fluorescence of ThT in these solvents, in an attempt to obtain narrower bands associated to a subset of conformations with uniform emissive properties. The synchronous technique is commonly employed when such band overlapping prevents the use of the traditional steady-state method [32]. The excitation and emission wavelengths are scanned simultaneously at a constant Δλ = λ_f_ − λ_ex_, which offers the advantages of spectral simplification and bandwidth reduction if Δλ is set to the Stokes shift of one of the spectral constituents [33]. Selected synchronous emissions of ThT in glycerol, at Δλ = 60 nm and 100 nm, which have been identified as the optimal values affording the best separation of the short and long wavelength emissions, are shown in Figure S5.

### 2.2. Gelation Behaviour of Pluronic F127/Polysaccharide Systems

The first step of the gelation study is to record the room temperature absorption, excitation and emission spectra of ThT in the three systems to be investigated: Pluronic F127 16.6% (P16.6%), Pluronic F127 16.6% in the presence of alginate 2% (P16.6%ALG2%) and Pluronic F127 16.6% in the presence of hyaluronic acid 0.5% (P16.6%HA0.5%). Then, the spectral behaviour of ThT upon heating in the temperature range 10–70 °C is analysed.

#### 2.2.1. Gelation of Pluronic F127

**Figure 1:**
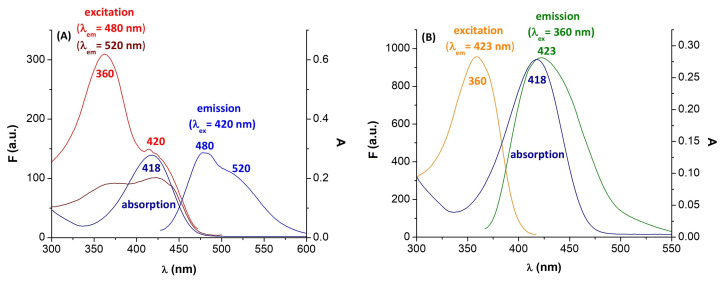
Absorption spectrum, excitation spectra recorded at λ_em_ = 423 nm (**A**), 480 and 520 nm (**B**) and steady-state fluorescence spectra recorded at λ_ex_ = 360 nm (**A**) and 420 nm (**B**) for ThT in P16.6%.

We firstly discuss the results obtained for the Pluronic F127 sample in the absence of polysaccharides. This is our reference sample with known gelation behaviour [16,17]. The absorption, excitation and emission spectra of ThT in P16.6% are shown in Figure 1. We can observe the presence of a single absorption band with a maximum at 418 nm. Excitation at this wavelength (~420 nm) results in the long wavelength emission with maxima at 480 and 520 nm (Figure 1A). The corresponding excitation spectra, obtained at either λ_f_ = 480 nm or 520 nm, are identical, with a lower intensity in the case of the latter. In the excitation spectra, a band at 360 nm can be easily observed alongside the band at 420 nm. As expected, based on the results of our solvatochromic study, excitation at 360 nm results in intense short wavelength emission at 423 nm (Figure 1B). Several Δλ values in the range 50–100 nm have been scanned, and the optimal values selected for the synchronous measurements, ensuring the maximal separation of emission bands, are Δλ = 50 nm (for the subset of conformers with λ_ex_ = 420 nm and λ_f_ = 480 nm), Δλ = 65 nm (for the conformers with λ_ex_ = 360 nm and λ_f_ = 423 nm) and Δλ = 100 nm (for the conformers with λ_ex_ = 420 nm and λ_f_ = 520 nm).

**Figure 2:**
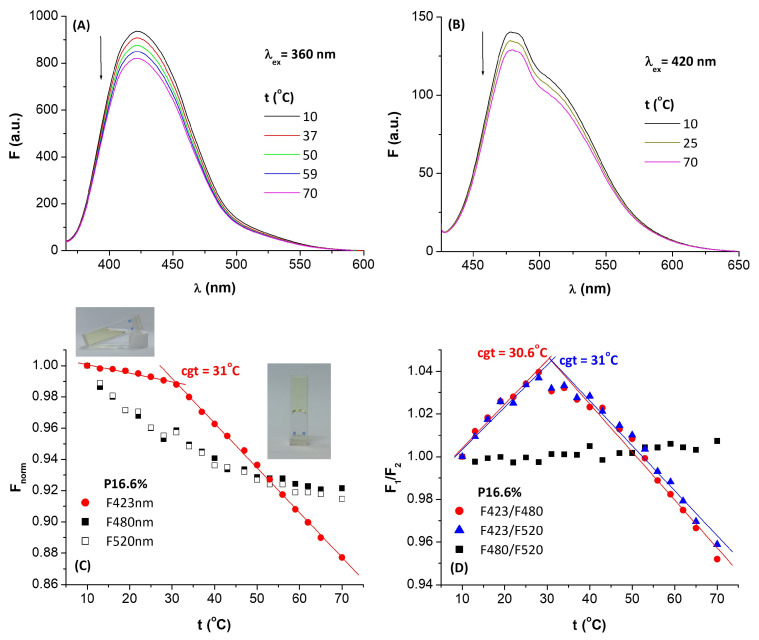
Temperature dependence of the steady-state fluorescence spectra of ThT in P16.6% recorded at λ_ex_ = 360 nm (**A**) and at λ_ex_ = 420 nm (**B**). Estimation of the critical gelation temperature (cgt) of P16.6% using the steady-state fluorescence intensity of ThT (**C**) and the ratio of ThT steady-state fluorescence intensities (**D**).

The spectral behaviour of ThT in the P16.6% sample, upon heating in the temperature range 10–70 °C, is depicted in Figure 2 and Figure S6. A decrease in both short and long wavelength emissions is observed with increasing temperature. This is due to the nonradiative deactivation pathways of the excited state that become favoured at higher temperatures [34]. The steeper decrease in the short wavelength steady-state emission at 423 nm (Figure 2A) as compared to the long wavelength emission (Figure 2B) correlates to the steeper decrease exhibited by the synchronous fluorescence intensity at 423 nm (Figure S6B) and is indicative of the fact that the population of ThT conformers changes with temperature.

Figure 2C and Figure S7A include plots of the normalized steady-state and synchronous fluorescence intensities as a function of temperature. No apparent change associated to the micellization of P16.6% is observed in these plots. According to our μDSC results (vide infra), the critical micelle temperature (cmt) of P16.6% is 13.0 °C. Nevertheless, the plots of the fluorescence intensity (either steady-state or synchronous) at 423 nm as a function of temperature present an evident change of slope associated to the micelle-to-gel transition. This break in the typical linear behaviour can be used to estimate the value of the critical gelation temperature (cgt) of P16.6%. Hence, the cgt is determined as the intersection point of two straight lines obtained by linearly fitting the data on the temperature domains corresponding to the micellar (lower temperatures) and gel (higher temperatures) phases. This method was applied successfully in previous studies [16,17] to estimate the cgt of P16.6% in the absence and in the presence of 2-hydroxypropyl-β-cyclodextrin from steady-state fluorescence and EPR data provided by dual molecular probes; the cgt value obtained with spectroscopic and rheological measurements was 31 °C. This value coincides to the cgt determined in this study, demonstrating that the ThT fluorescence method proposed here constitutes a suitable means for accurately determining the gelation temperature of Pluronic F127. The steady-state and synchronous techniques are consistent, yielding the same result.

We also paid attention to the temperature dependence of the ratios of fluorescence intensities at 423/480, 423/520 and 480/520 nm, which correlate to changes in the population of conformers during heating and gelation. From the plots shown in Figure 2D, we observe that the ratios involving the steady-state emission at 423 nm are indeed responsive to gelation, yielding a cgt value of ~31 °C. The synchronous data, due to very small changes in band intensities, show more noise and cannot be accurately used to estimate cgt in this case (Figure S7B).

**Figure 3:**
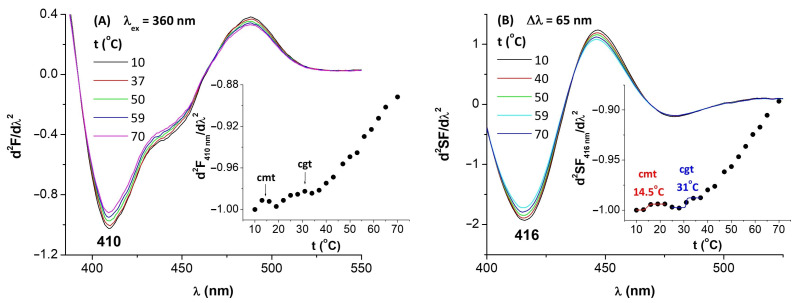
Second-derivative steady-state fluorescence (**A**), λ_ex_ = 360 nm, and second-derivative synchronous fluorescence (**B**), Δλ = 65 nm, spectra of ThT in P16.6%. Inset of (**A**): Plot of the second-derivative fluorescence intensity (normalized) at 410 nm as a function of temperature. Inset of (**B**): Determination of the cmt and cgt values from the plot of the second-derivative synchronous fluorescence intensity (normalized) at 416 nm as a function of temperature. The solid lines represent the best fits of the data to sigmoid functions.

Finally, we tested the utility of using the second derivative of the steady-state and synchronous fluorescence of ThT to extract supplementary information on the system. Applying the differentiation process to the steady-state fluorescence emissions at 480 and 520 nm results in a monotonous increase in the second-derivative fluorescence intensity, without any inflection point, rendering impossible the determination of cgt. Differently, in the case of the steady-state fluorescence at 423 nm (Figure 3A, corresponding minimum at 410 nm in the second-derivative spectrum), the plot of d^2^SF_410nm_/dλ^2^ as a function of temperature shows distinct features around 15 °C and 30 °C that can be associated to the micellization process and to the micelle-to-gel transition of P16.6%, respectively (inset of Figure 3A). The inflection points become most evident in the second derivative of the synchronous fluorescence at 423 nm (Figure 3B, corresponding minimum at 416 nm). Fitting this data to sigmoid functions in the temperature domains 10–22 °C, corresponding to micellization, and 25–37 °C, corresponding to gelation, allows us to successfully extract the values of the cmt = 14.5 °C and cgt = 31 °C of P16.6% (inset of Figure 3B).

In the following, we present our results on the gelation behaviour of Pluronic F127/polysaccharide systems and comment on the suitability of ThT as a fluorescent probe for these gelling systems.

#### 2.2.2. Gelation of Pluronic F127/Alginate

**Figure 4:**
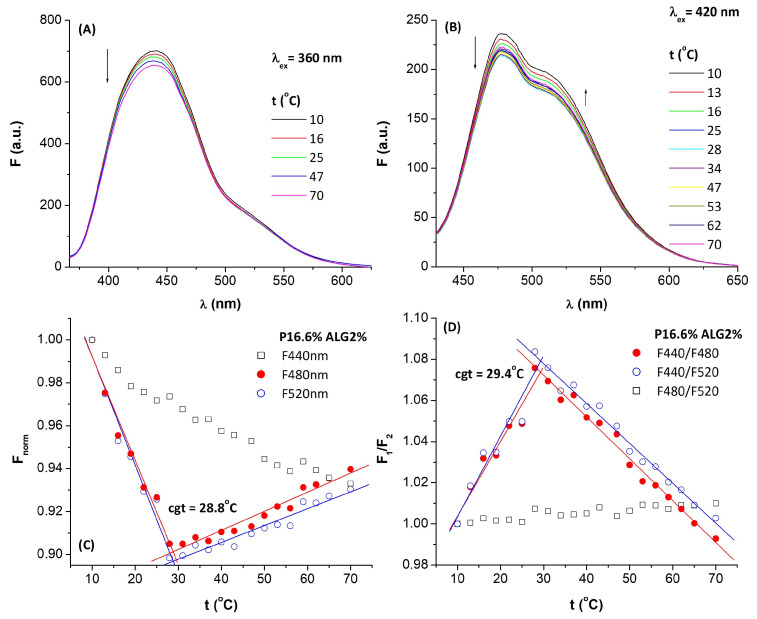
Temperature dependence of the steady-state fluorescence spectra of ThT in P16.6%ALG2% recorded at λ_ex_ = 360 nm (**A**) and at λ_ex_ = 420 nm (**B**). Estimation of the critical gelation temperature (cgt) of P16.6%ALG2% using the steady-state fluorescence intensity of ThT (**C**) and the ratio of ThT steady-state fluorescence intensities (**D**).

The first polysaccharide selected in this study is alginate. The absorption, excitation and steady-state fluorescence spectra of ThT in the P16.6%ALG2% sample are shown in Figure S8. They are similar to those displayed for ThT in P16.6%, with the exception of an ~20 nm bathochromic shift to 440 nm of the short wavelength emission. Differently from the case of P16.6%, a steeper decrease is now observed for the long wavelength steady-state emission at 480 and 520 nm (Figure 4). Moreover, the initial fluorescence decrease is followed by a stationary response at temperatures between 28 and 34 °C, then by a fluorescence increase. An increase in the fluorescence intensity of ThT, overcoming the quenching effect exerted by the temperature increase, could be rationalized in terms of an enhancement in the rigidity of the microenvironment [14] due to the formation of a stronger gel network in the presence of alginate. Indeed, rheological measurements evidenced significant increase in gel strength by combining Pluronic F127 with alginate [35], due to water molecules acting as “crosslinkers” to form hydrogen bonds between the ether groups of Pluronic F127 and the carboxyl groups of the alginate.

Plotting the intensities of the steady-state fluorescence bands as a function of temperature, we observe in the case of the long wavelength emission at 480 nm and 520 nm the existence of two linear domains between 10–28 °C and 28–70 °C (Figure 4C). The intersection of the corresponding linear fits yields, in both cases, a cgt value of 28.8 °C, which is lower than the cgt value of P16.6%. Interestingly, in the presence of alginate, the short wavelength emission of ThT is only weakly responsive to temperature changes and cannot be used to estimate the cgt. For the P16.6%ALG2% system, the ThT conformers with planar and quasi-planar geometry, responsible for the long wavelength emission, serve as reporters of gelation, and this might be due to their easier insertion within the more tightly packed gel network.

For synchronous experiments, we used Δλ = 50 nm (for the subset of conformers with λ_ex_ = 420 nm and λ_f_ = 480 nm) and Δλ = 80 nm (for conformers with λ_ex_ = 360 nm and λ_f_ = 440 nm and with λ_ex_ = 420 nm and λ_f_ = 520 nm) (Figure S9). A similar trend to that observed via the steady-state technique emerges from the synchronous data, with an estimated cgt value of ~29 °C (Figure S10A).

When using the temperature dependence of the ratios of fluorescence intensities at 440/480, 440/520 and 480/520 nm (Figure 4D and Figure S10B), only the ratios involving the long wavelength emission at either 480 or 520 nm are responsive to gelation, as expected. The estimated cgt values from steady-state and synchronous data coincide and are slightly larger (29.4 °C).

No effect of gelation on the second derivative of the steady-state fluorescence bands at 440, 480 and 520 nm was observed. In the case of the second derivative of the synchronous fluorescence at 440 nm, the plot d^2^SF_440 nm_/dλ^2^ (Figure S11) shows marginal changes corresponding to micellization (10–13 °C) and gelation (25–28 °C). Micellization occurs at lower temperatures as compared to P16.6%, a trend that is confirmed by the μDSC results (cmt = 11.6 °C, vide infra). Gelation also occurs at lower temperatures, as revealed in the results presented above.

#### 2.2.3. Gelation of Pluronic F127/Hyaluronic Acid

**Figure 5:**
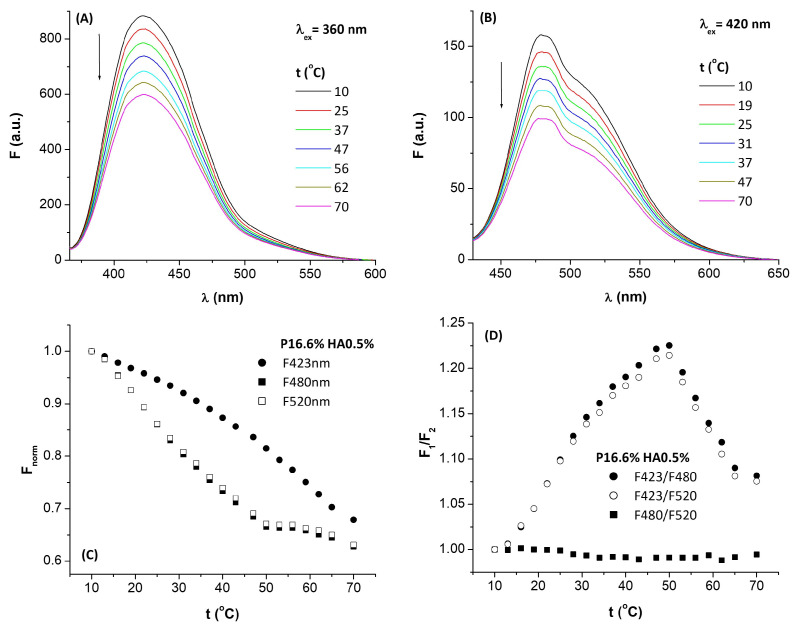
Temperature dependence of the steady-state fluorescence spectra of ThT in P16.6%HA0.5% recorded at λ_ex_ = 360 nm (**A**) and at λ_ex_ = 420 nm (**B**). Plots of the steady-state fluorescence intensities of ThT (**C**) and of the ratios of ThT steady-state fluorescence intensities (**D**) as a function of temperature.

The second polysaccharide selected for our study is hyaluronic acid. Although the spectral features of ThT in the P16.6%HA0.5% sample (Figure S12) closely resemble those of ThT in P16.6%, the spectral behaviour of ThT during heating is markedly different from that in P16.6% or in P16.6%ALG2%, pointing to different interactions occurring in the P16.6%HA0.5% system. As can be seen from Figure 5, ThT displays the most notable fluorescence decrease in both the short wavelength and the long wavelength emissions. The synchronous spectra acquired at Δλ = 50 nm (for the subset of conformers with λ_ex_ = 420 nm and λ_f_ = 480 nm), at Δλ = 65 nm (for the conformers with λ_ex_ = 360 nm and λ_em_ = 423 nm) and at Δλ = 90 nm (for the conformers with λ_ex_ = 420 nm and λ_em_ = 520 nm) display a significant intensity decrease as well (Figure S13).

Surprisingly, the plots of the steady-state fluorescence (Figure 5C) and synchronous fluorescence (Figure S14A) intensities as a function of temperature do not reveal the same striking change in slope around the gelation temperature as seen in the case of P16% and P16%ALG2%. A break in the monotonous variation is observed at ~50 °C, and it has yet to be explained. As the μDSC scans display no transition at this temperature, a local change sensed via the ThT probe rather than a strong, global one could cause the break observed at 50 °C.

**Figure 6:**
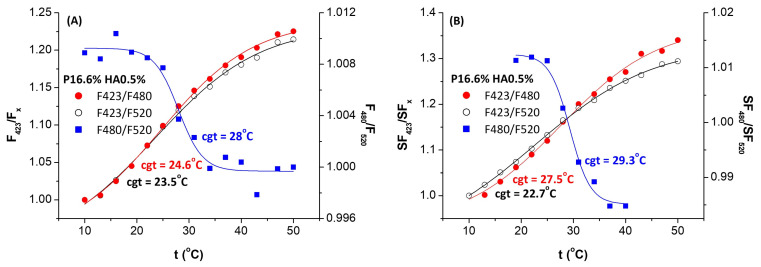
Estimation of the critical gelation temperature (cgt) of the P16.6%HA0.5% system from the ratios of steady-state fluorescence (**A**) and synchronous fluorescence (**B**) intensities. The solid lines represent the best fits of the data to sigmoid functions.

When analysing the temperature dependence of the ratios of fluorescence intensities at 423/480, 423/520 and 480/520 nm (Figure 5D and Figure S14B), we observe that the data in the temperature domain 10–50 °C show a gradual variation that can be fitted to a sigmoidal function, allowing estimation of the cgt (Figure 6). This method yields quite different cgt values, in the range 22.7–29.3 °C, depending on the band ratio considered and the technique used (steady-state vs. synchronous). Lower cgt values are obtained when the short wavelength emission is considered, while higher cgt values are determined when considering the long wavelength emission. We believe that these differences might be due to the stabilization of different ThT conformers in different regions of the gelling systems, each reporting from their respective microenvironment. A decrease in the gelation temperature in the presence of high molecular weight hyaluronic acid has been explained in the literature by the insertion of hyaluronic chains in the interstitial spaces between closely packed Pluronic F127 micelles. In this way, the interaction between Pluronic chains and water molecules is hampered, which hastens the assembly of micelles into gel [36].

In the case of the P16.6%HA0.5% system, the second-derivative steady-state or synchronous spectra do not provide additional information on micellization or gelation. The cmt value obtained from μDSC measurements is 12.6 °C (vide infra).

**Figure 7:**
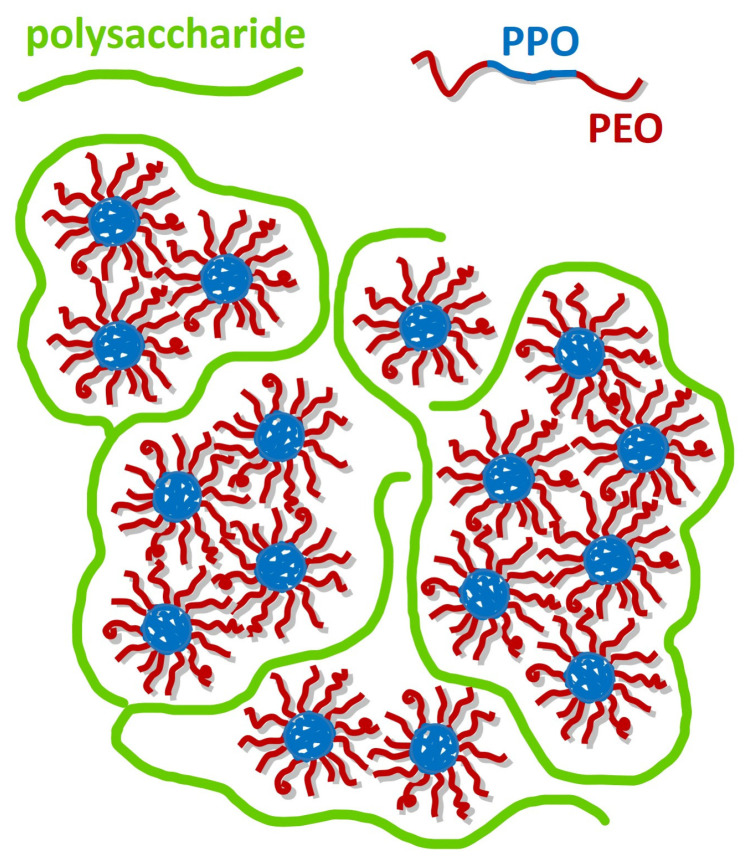
Schematic representation of the Pluronic F127/polysaccharide systems. The Pluronic F127 subdomains, containing micelles with interpenetrated coronae, are caged by polysaccharide chains.

The results discussed above point to the presence of several ThT conformers, with temperature-dependent populations, which become entrapped in different regions of the Pluronic F127/polysaccharide systems. Their spectral response is dictated by their localization, either within the F127 micellar cores or coronae, near the negatively-charged polysaccharide chains or in the solvent pools (please refer to Figure 7 for a schematic representation of the Pluronic F127/polysaccharide systems, in accordance to the interaction mechanisms proposed in the literature for these hydrogels [20,36]). It would be interesting to explore further the versatility of ThT as a fluorescent reporter in other gelling systems that involve different types of supramolecular interactions. Spectroscopic methods like the one proposed here are essential for a thorough characterization at the molecular level of heterogeneous systems involving continuous molecular reorganization and gradual changes in the hydrophobic/hydrophilic balance. These methods rely on the adequate selection of molecular probes, and we believe ThT is a good candidate.

### 2.3. Differential Scanning Microcalorimetry (μDSC) Analysis

In the following section, the effect of the two polysaccharides, alginate and hyaluronic acid, on the micellization process of Pluronic P16.6% is evaluated at macroscopic level using μDSC measurements. Gelation, which is a weak endothermic transition involving micelle growth and aggregation, is not noticed with μDSC [21].

**Figure 8:**
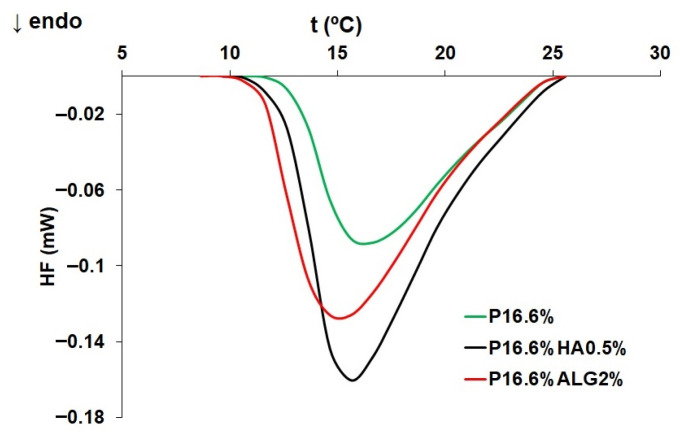
The μDSC scans of P16.6% micellization, in the absence and in the presence of HA0.5% and ALG2%.

It is known that, at low temperatures, Pluronic F127 exists in a disordered state as individual coils (unimers). Above the critical micelle concentration (cmc) and the cmt [37], this block copolymer self-assembles into spherical micelles with the hydrophobic PPO blocks as the core [38] and the hydrophilic PEO blocks as the corona [39]. The μDSC scans (Figure 8) present endothermic signals corresponding to the micellization of Pluronic F127 caused by the aggregation of Pluronic F127 unimers into micellar structures [40].

The cooling μDSC scans (Figure S15) display exothermic peaks assigned to the micelle melting process, which occurs with the temperature decrease. Analyzing heating and cooling scans of P16.6% in the absence and in the presence of HA0.5% or ALG2%, it can be observed that the endothermic and exothermic peaks are symmetric, displaying the thermal equilibrium of micelle formation, as previously reported [41].

**Figure 9:**
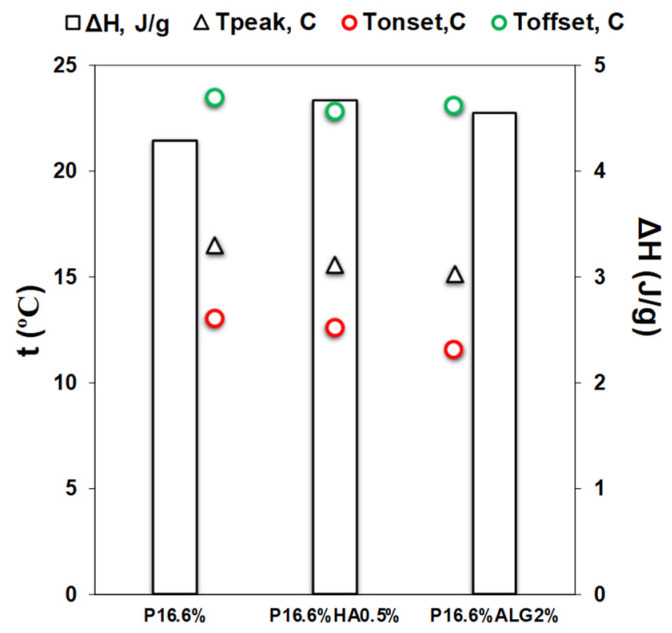
Thermodynamic parameters of P16.6% micellization, in the absence and in the presence of HA0.5% and ALG2%.

The incorporation of drugs or additives to aqueous Pluronic dispersions influences the cmc, cmt and the structure of the micelles [42,43]. The thermodynamic parameters of micellization, and the effects of HA0.5% and of ALG2% on the P16.6% micellization process are presented in Figure 9.

The values of the micellization temperatures and enthalpy for P16.6% are in good agreement with data reported in the literature [44]. Figure 9 shows that the onset of the endothermic peak (T_onset_ considered as cmt), T_peak_ and T_offset_ shift to lower temperatures in the presence of HA0.5% and ALG2%, indicating a favorable effect on the Pluronic F127 micellization process. Previous studies reported lower values for cmt of Pluronic F127 in interaction with cellulose derivatives, as result of hydrogen bonding between cellulose derivatives and PEO blocks of F127 that decreased the effective block length of PEO, thereby facilitating micellization [45]. Also, the addition of poly (acrylic acid) to Pluronic F127 decreases the cmt of F127 due to association between the ether oxygens of PEO in F127 and the carboxylic groups of poly(acrylic acid) via hydrogen bonding [46]. Some P16.6% chains may be associated with HA0.5% and ALG2%, leading to the formation of aggregates at a lower temperature. Moreover, the increase in the micellization enthalpy (ΔH) observed in the presence of HA0.5% and ALG2% is the result of the aggregation of a larger number of Pluronic molecules [47], suggesting that both polysaccharides exhibit micellization-promoting behaviour. As a result of the interaction between Pluronic micelles and polysaccharides, the presence of HA0.5% and ALG2% further influences the gelation process of P16.6% [48,49], modifying the cgt value, as demonstrated by the fluorescence spectroscopy results discussed above.

## 3. Conclusions

This investigation has shown that the fluorescence response of the ThT probe is a suitable method for determining critical gelation temperatures, based on temperature-induced changes in the steady-state and synchronous emissions of ThT.

For Pluronic F127 and the Pluronic F127/polysaccharide systems under investigation, the determination of the cgt value can be made either via steady-state or synchronous fluorescence. The data obtained are in line with reports in the literature, evidencing a decrease in the temperature of the micelle-to-gel phase transition in the presence of polysaccharides. Namely, the cgt value decreases from 31 °C (in P16.6%) to 28.8–29.4 °C (in P16.6%ALG2%) and to 22.7–29.3 °C (in P16.6%HA0.5%). These variation intervals can be explained by the heterogeneity of the Pluronic F127/polysaccharide systems favouring the presence of several ThT conformers, from planar/quasi-planar conformers to twisted conformers with a broken π-electron system. Heating produces changes in the conformer populations as they become entrapped in different regions of the gelling systems, from which they act as reporters of the respective microenvironment.

Differentiation of the emission spectra is not necessary for the estimation of the cgt values, but the second derivative of the fluorescence or synchronous fluorescence spectra can provide additional insight regarding the micellization domain, as confirmed by correlating with μDSC data. While the fluorimetric method has allowed us to explore the micelle-to-gel phase transition in Pluronic/polysaccharide systems at the molecular level, the μDSC measurements have provided complementary macroscopic insights into the micellization process, e.g., the thermodynamic parameters of micellization and the effect of HA0.5% and of ALG2% on the P16.6% micellization process. The presence of polysaccharides promotes the micellization of Pluronic F127. The T_onse_t (cmt) value slightly decreases from 13.0 °C to 12.6 °C in the presence of HA0.5%, while ALG2% induces a more pronounced decrease in the cmt value to 11.6 °C. The same effect was observed for the T_peak_ and T_offset_ values. The T_peak_ value of P16.6% (16.5 °C) shifts to lower temperatures in the presence of HA0.5% (15.5 °C) and ALG2% (15.1 °C), while the T_offset_ value decreases from 23.5 °C for P16.6% to 22.8 °C in the presence of HA0.5% and 23.1 °C in the presence of ALG2%

We consider that the ThT-based fluorimetric method proposed here could be extended to other types of gelling systems. As such, this study can be of interest to researchers working in the fields of spectroscopy and supramolecular gels.

## 4. Materials and Methods

Thioflavin T, Pluronic F127 (molecular weight 12.6 kDa, PEO:PPO ~ 4.1:1), glycerol diethylene glycol and methanol were obtained from Sigma Aldrich (St. Louis, MO, USA). Low viscosity alginic acid sodium salt was purchased from Alfa Aesar (Ward Hill, MA, USA). Hyaluronic acid sodium salt (95%, molecular weight 1500–2200 kDa) was from Acros Organics (Geel, Belgium).

Samples were prepared at room temperature by dissolving Pluronic F127 (16.6% wt.) in distilled water, in the absence (sample P16.6%) or in the presence of 2% wt. alginate (sample P16.6%ALG2%) or 0.5% wt. hyaluronic acid (sample P16.6%HA0.5%). The Pluronic F127 and polysaccharide concentrations for the model systems were selected by surveying the literature data regarding their optimal concentrations for pharmaceutical applications [22,35]. The 16.6% concentration of Pluronic F127 is close to the lowest concentration at which the micelle-to-gel transition can be observed [50]. All samples were kept overnight at 8 °C, then subjected to thermal treatment in the temperature range 10–60 °C. The concentration of Thioflavin T in the samples was 10^−5^ M. Two blank samples containing only alginate 2% wt. and only hyaluronic acid 0.5% wt. were subjected to the same thermal treatment (Figure S16) in order to discard any interference from the polysaccharide component on the variation of the ThT emission in the Pluronic F127/polysaccharide systems.

Electronic absorption spectra were recorded on a V-560 JASCO UV−Vis spectrophotometer (JASCO Co., Ltd., Kyoto, Japan) at 25 °C.

Steady-state fluorescence, synchronous fluorescence and excitation spectra were recorded on a JASCO FP-6500 spectrofluorometer (JASCO Co., Ltd., Kyoto, Japan). The temperature was varied in the range 10–70 °C with a step of 3 °C. Each sample was kept for 20 min at each temperature to ensure equilibration prior to recording the spectrum.

The effect of HA0.5% and ALG2% on the micellization process of Pluronic P16.6% was evaluated by μDSC measurements using an μDSC7evo instrument (Setaram, Caluire, France) in the temperature range 1–70 °C, with 1 °C min^−1^ scan rate. The reversibility of micellization was checked by cooling down the samples to 1 °C. Data acquisition and processing were performed using Calisto v.1077, the software supplied by the instrument manufacturer, with a linear baseline. The T_onset_, T_peak_, T_offset_ and the micellization enthalpy (ΔH) of P16.6% in the absence and in the presence of HA0.5% and ALG2% were determined. T_onset_ corresponds to the cmt as the temperature where micelles start to form, while T_offset_ corresponds to the temperature where the micellization is complete. T_peak_ represents the temperature of the maximum heat flow. ΔH was determined from the area by integrating the endothermic peak [51,52].

## Data Availability

Data are contained within the article and the Supplementary Material.

## Supplementary Materials

The following supporting information can be downloaded at: https://www.mdpi.com/article/10.3390/gels9120939/s1, Figure S1: Steady-state fluorescence spectra of ThT in glycerol at increasing concentrations in the range of 10^−6^–10^−4^ M. The excitation/emission wavelengths are marked on the spectra. All spectra are recorded in the same conditions; Figure S2: Excitation spectra of ThT in glycerol at different concentrations. The excitation/emission wavelengths are marked on the spectra; Figure S3: Steady-state fluorescence spectra of ThT in diethylene glycol and methanol at different concentrations. The excitation/emission wavelengths are marked on the spectra. All spectra are recorded in the same conditions; Figure S4: Excitation spectra of ThT in diethylene glycol and methanol at different concentrations. The excitation/emission wavelengths are marked on the spectra; Figure S5: Synchronous fluorescence spectra of ThT in glycerol at increasing concentrations in the range of 10^−6^–10^−4^ M. The excitation/emission pairs are marked on the spectra; Figure S6: Temperature dependence of the synchronous fluorescence spectra of ThT in P16.6% obtained at Δλ = 50 nm (A), 65 nm (B) and 100 nm (C); Figure S7: Estimation of the critical gelation temperature (cgt) of P16.6% using synchronous fluorescence data; Figure S8: Absorption spectrum, excitation spectra recorded at λ_em_ = 440, 480 and 520 nm and steady-state fluorescence spectra recorded at λ_ex_ = 360 and 420 nm for ThT in P16.6%ALG2%; Figure S9: Temperature dependence of the synchronous fluorescence spectra of ThT in P16.6%ALG2%: (A) Δλ = 50 nm, (B) Δλ = 80 nm; Figure S10: Estimation of the critical gelation temperature (cgt) of P16.6%ALG2% using the synchronous fluorescence emission (A) and the ratio of ThT synchronous fluorescence emissions (B); Figure S11: Second-derivative synchronous fluorescence (Δλ = 80 nm) spectra of ThT in P16.6%ALG2%. Inset: Plot of the second-derivative fluorescence intensity (normalized) at 440 nm as a function of temperature; Figure S12: Absorption spectrum, excitation spectra recorded at λ_em_ = 423, 480 and 520 nm and steady-state fluorescence spectra recorded at λ_ex_ = 360 and 420 nm for ThT in P16.6%HA0.5%; Figure S13: Temperature dependence of the synchronous fluorescence spectra of ThT in P16.6%HA0.5%: (A) Δλ = 50 nm, (B) Δλ = 65 nm and (C) Δλ = 90 nm; Figure S14: Plots of the synchronous fluorescence emission (A) and of the ratio of ThT synchronous fluorescence emissions (B) of ThT in P16.6%HA0.5% as a function of temperature; Figure S15: Heating and cooling μDSC scans of P16.6% in the absence and in the presence of HA0.5% or ALG2%; Figure S16: Reference plots for alginate (A—steady-state fluorescence intensities and B—ratio of steady-state fluorescence intensities) and hyaluronic acid (C—ratio of steady-state fluorescence intensities) showing no discontinuity at the gelation temperature.
