# Supplementary material for: Gelation Behaviour of Pluronic F127/Polysaccharide Systems Revealed via Thioflavin T Fluorescence
Source: Gels. 2023 Nov 30;9(12):939. doi: 10.3390/gels9120939 (PMC10742936; doi:10.3390/gels9120939)
Supplement: Supplementary file 1 [file gels-09-00939-s001.zip › gels-2720928-supplementary.pdf]

Supplementary Material

Gelation Behavior of Pluronic F127/Polysaccharide Systems Revealed by Thioflavin T Fluorescence

George-Alin Balan, Aurica Precupas, Iulia Matei *

"Ilie Murgulescu" Institute of Physical Chemistry of the Romanian Academy,
202 Splaiul Independentei, Bucharest 060021, Romania

***** Correspondence: iuliamatei@icf.ro

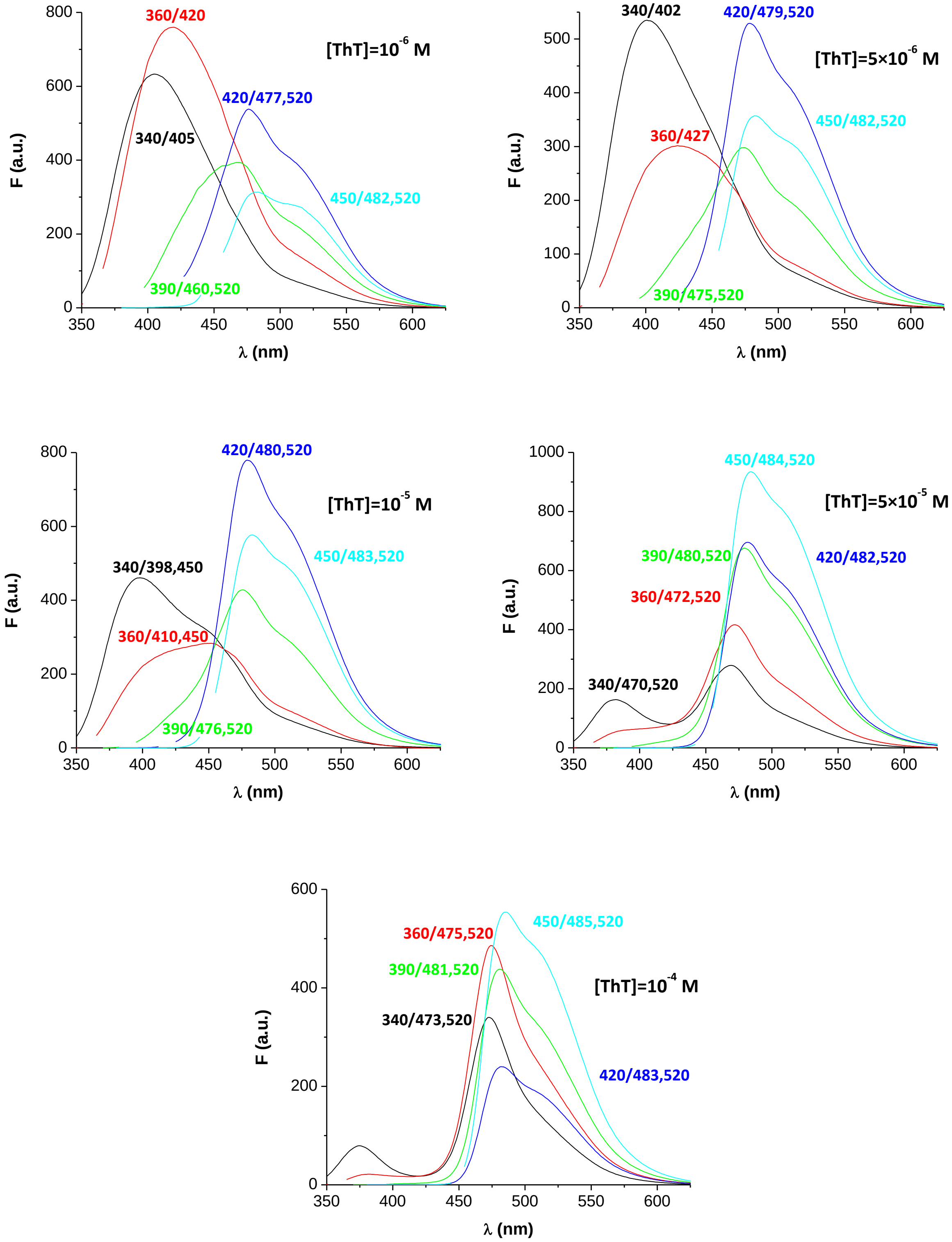

Figure S1. Steady-state fluorescence spectra of ThT in glycerol at increasing concentrations in the range 10^{-6}–10^{-4} M. The excitation/emission wavelengths are marked on the spectra. All spectra are recorded in the same conditions.

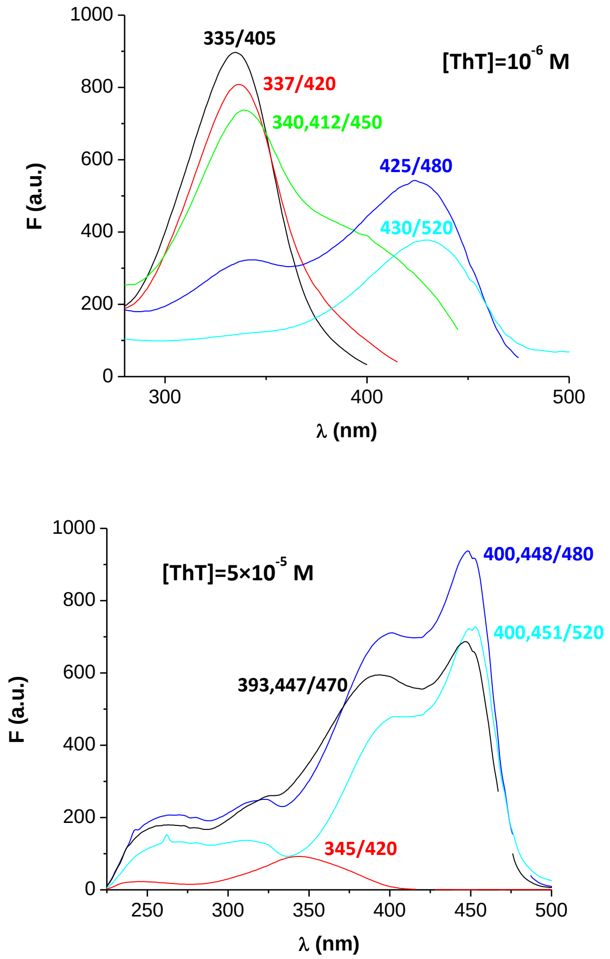

Figure S2. Excitation spectra of ThT in glycerol at different concentrations. The excitation/emission wavelengths are marked on the spectra.

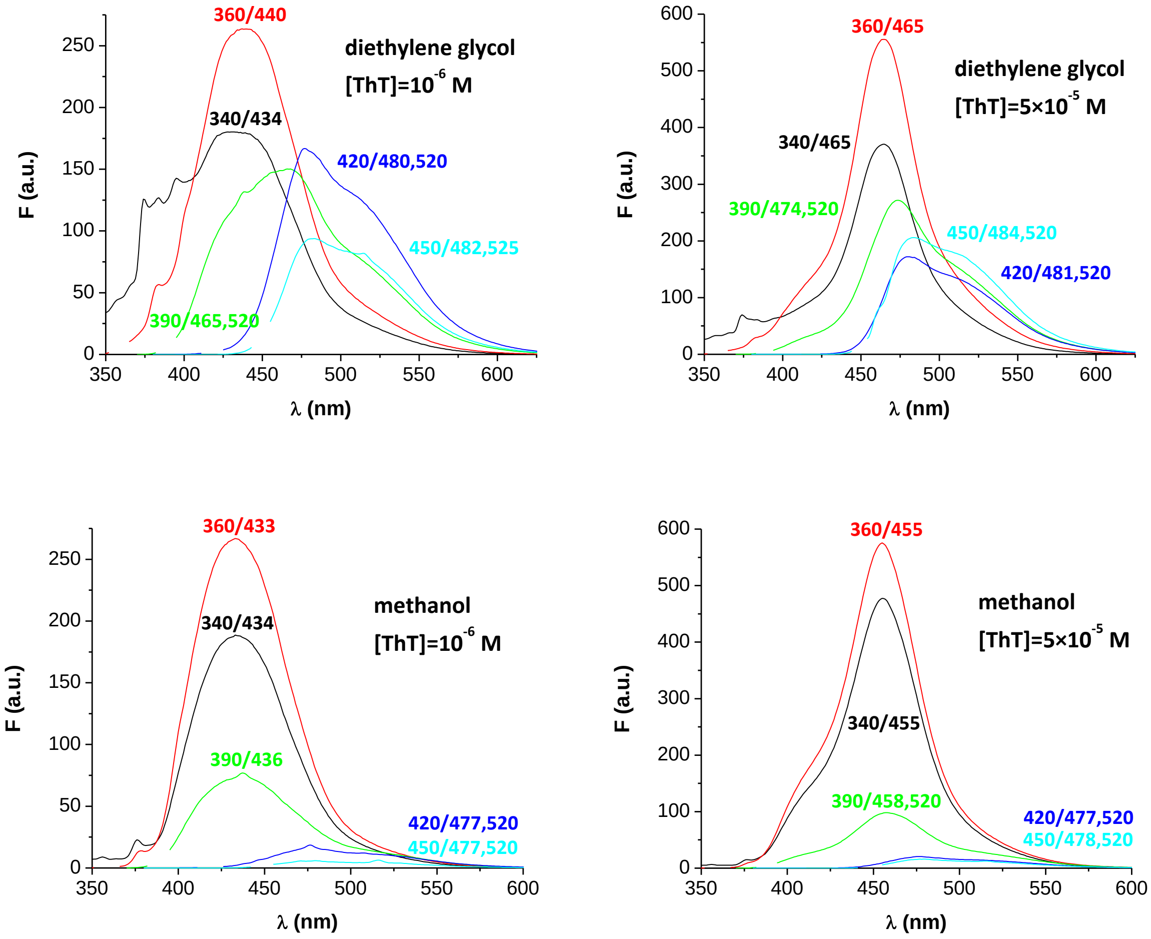

Figure S3. Steady-state fluorescence spectra of ThT in diethylene glycol and methanol at different concentrations. The excitation/emission wavelengths are marked on the spectra. All spectra are recorded in the same conditions.

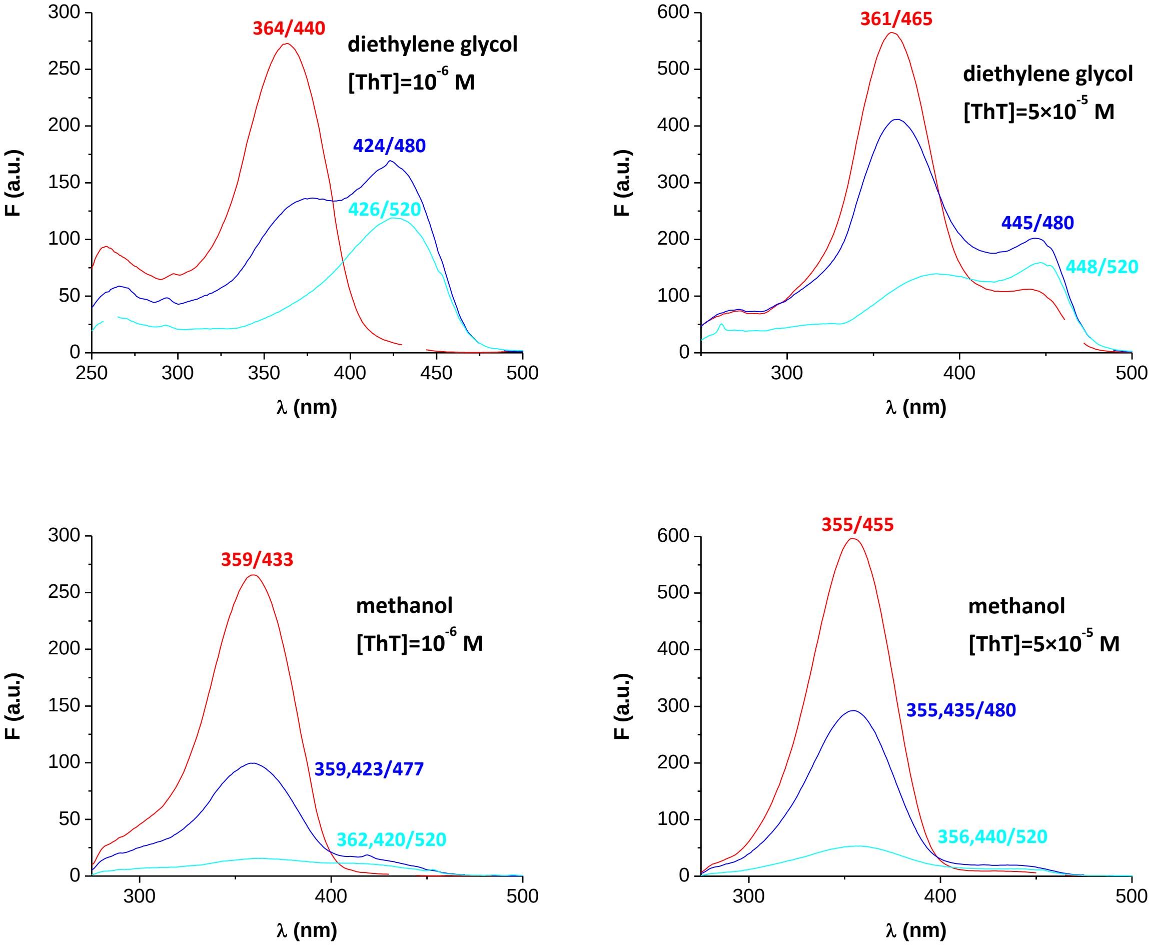

Figure S4. Excitation spectra of ThT in diethylene glycol and methanol at different concentrations. The excitation/emission wavelengths are marked on the spectra.

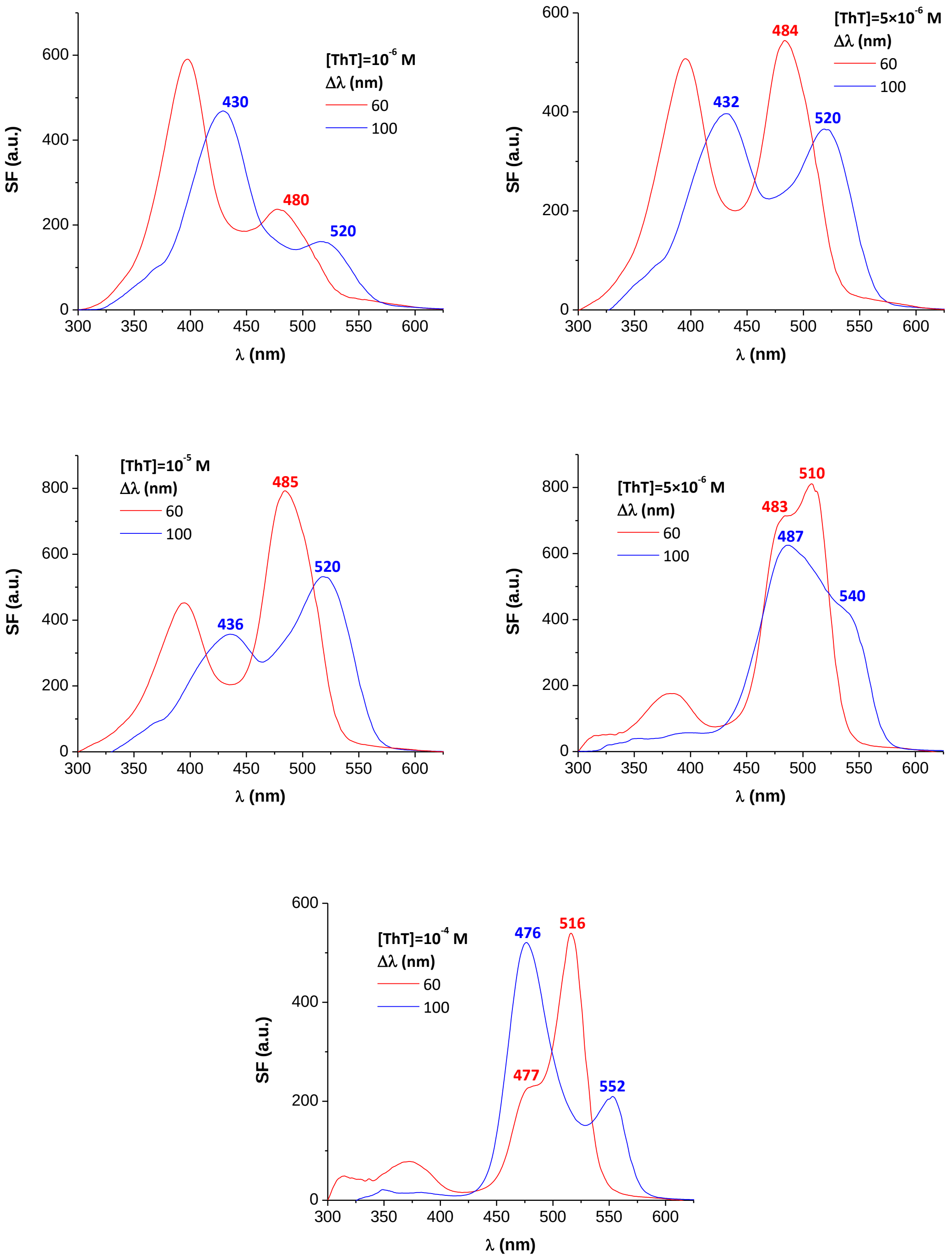

Figure S5. Synchronous fluorescence spectra of ThT in glycerol at increasing concentrations in the range 10^{-6}–10^{-4} M. The excitation/emission pairs are marked on the spectra.

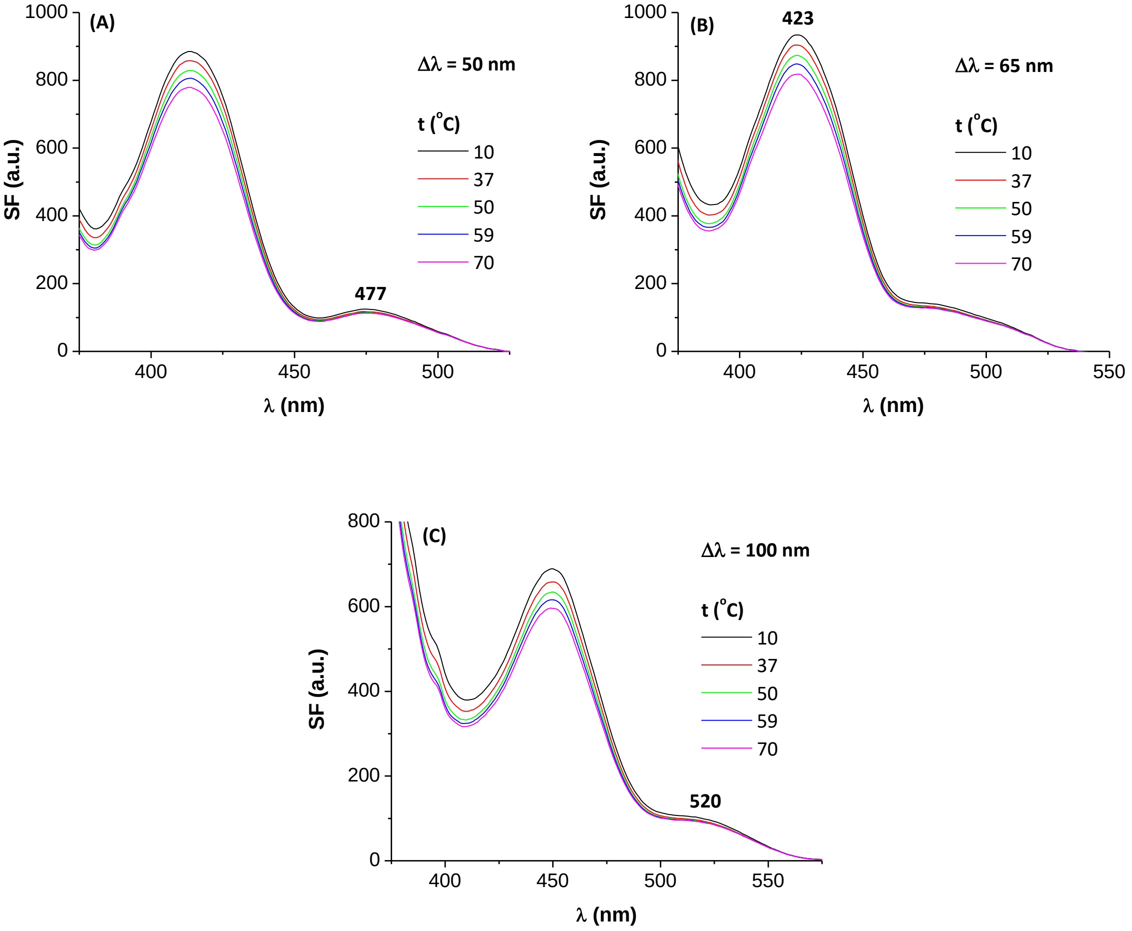

Figure S6. Temperature dependence of the synchronous fluorescence spectra of ThT in P16.6%: (A) Δλ = 50 nm, (B) Δλ = 65 nm, (C) Δλ = 100 nm.

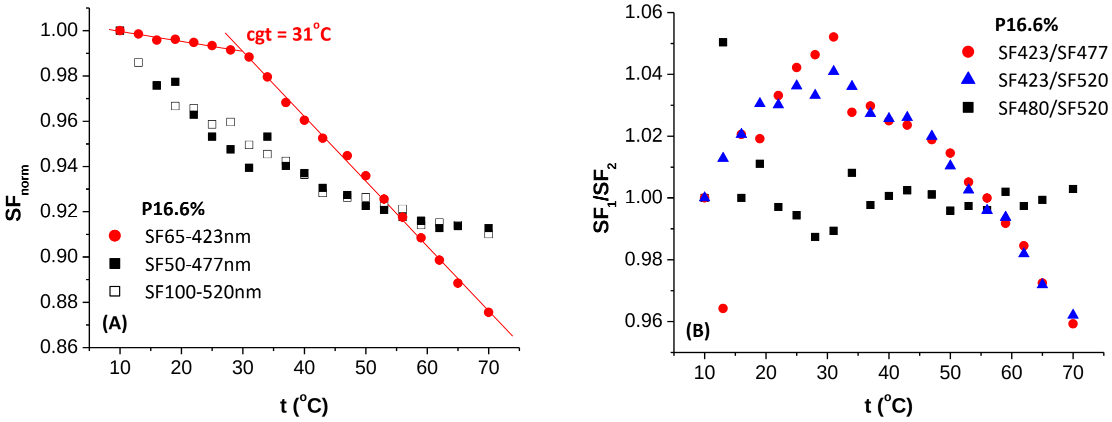

Figure S7. Estimation of the critical gelation temperature (cgt) of P16.6% using the synchronous fluorescence intensity (A) and the ratio of ThT synchronous fluorescence intensities (B).

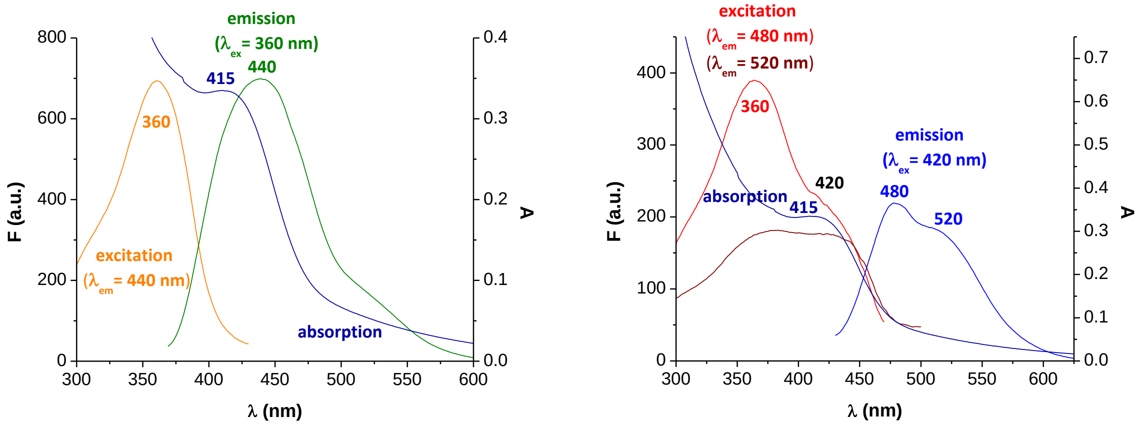

Figure S8. Absorption spectrum, excitation spectra recorded at λ_{em} = 440, 480 and 520 nm and steady-state fluorescence spectra recorded at λ_{ex} = 360 and 420 nm for ThT in P16.6%ALG2%.

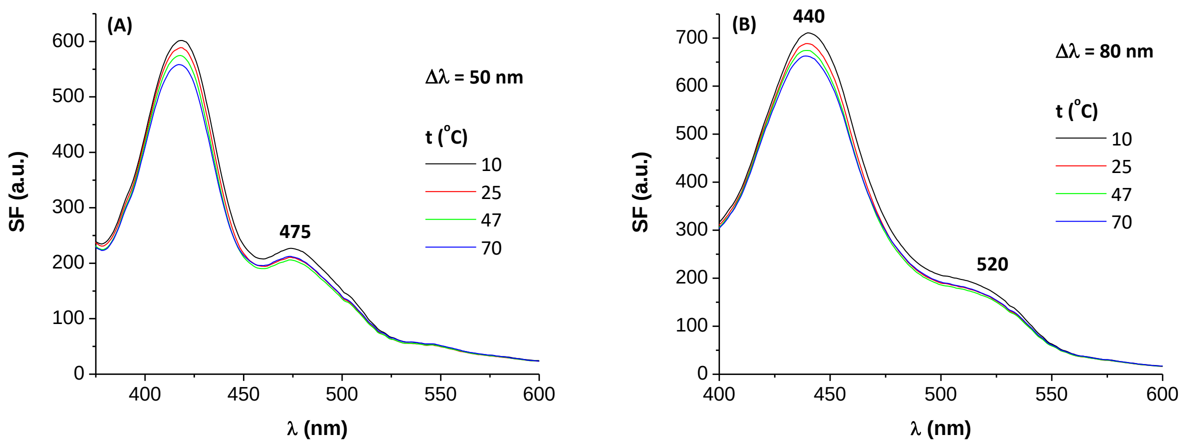

Figure S9. Temperature dependence of the synchronous fluorescence spectra of ThT in P16.6%ALG2%: (A) Δλ = 50 nm, (B) Δλ = 80 nm.

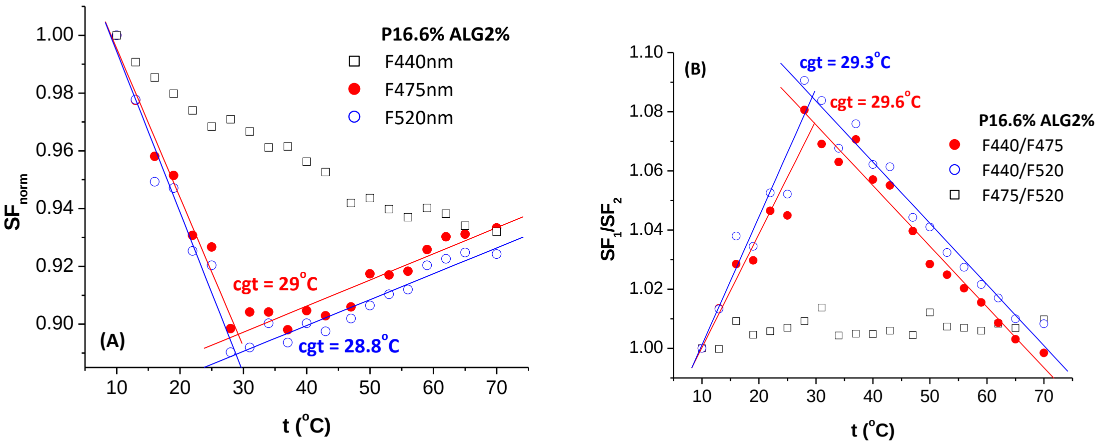

Figure S10. Estimation of the critical gelation temperature (cgt) of P16.6%ALG2% using the synchronous fluorescence intensity (A) and the ratio of ThT synchronous fluorescence intensities (B).

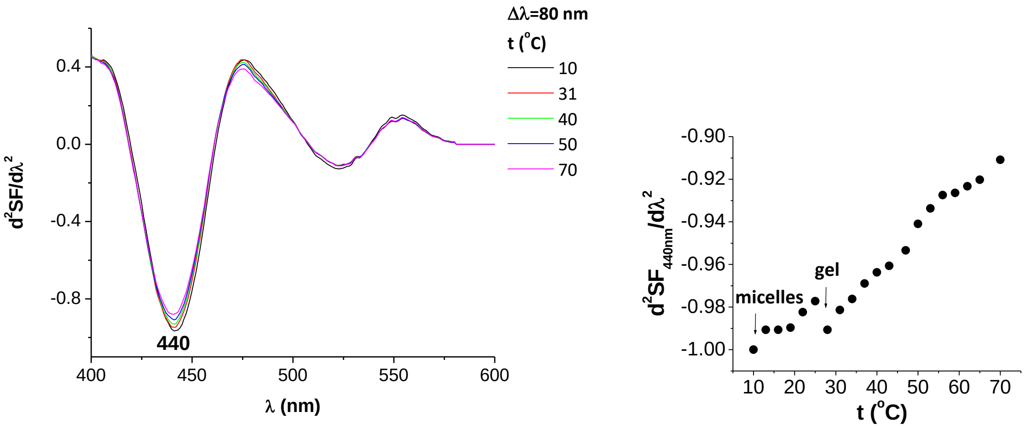

Figure S11. Second-derivative synchronous fluorescence (Δλ = 80 nm) spectra of ThT in P16.6%ALG2%. Inset: Plot of the second-derivative fluorescence intensity (normalized) at 440 nm as a function of temperature.

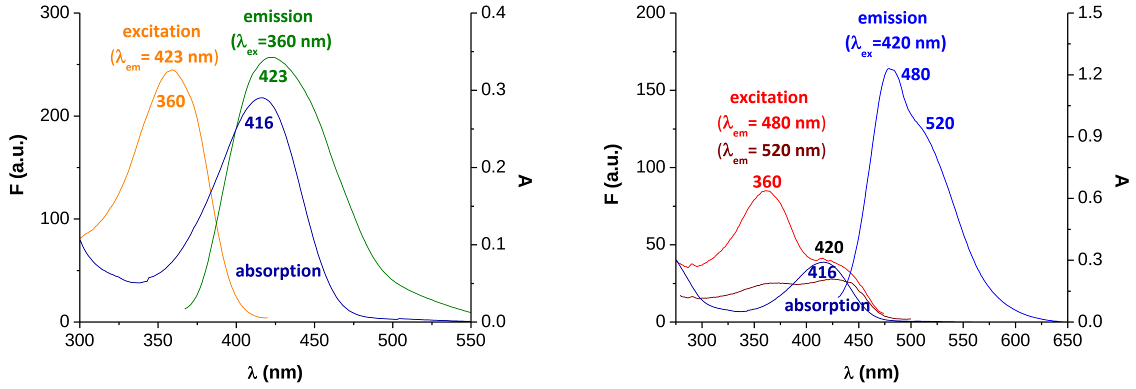

Figure S12. Absorption spectrum, excitation spectra recorded at λ_{em} = 423, 480 and 520 nm and steady-state fluorescence spectra recorded at λ_{ex} = 360 and 420 nm for ThT in P16.6%HA0.5%.

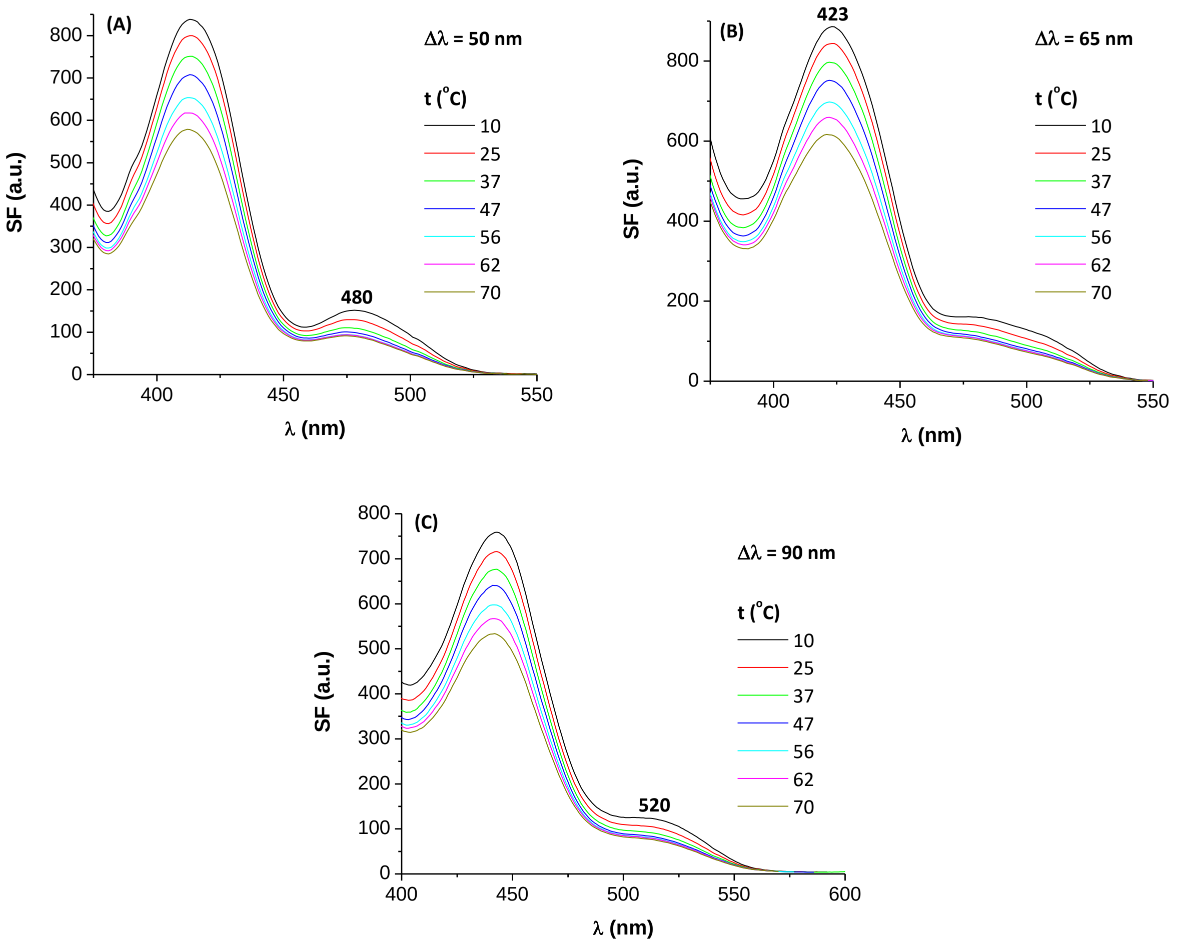

Figure S13. Temperature dependence of the synchronous fluorescence spectra of ThT in P16.6%HA0.5%: (A) Δλ = 50 nm, (B) Δλ = 65 nm, (C) Δλ = 90 nm.

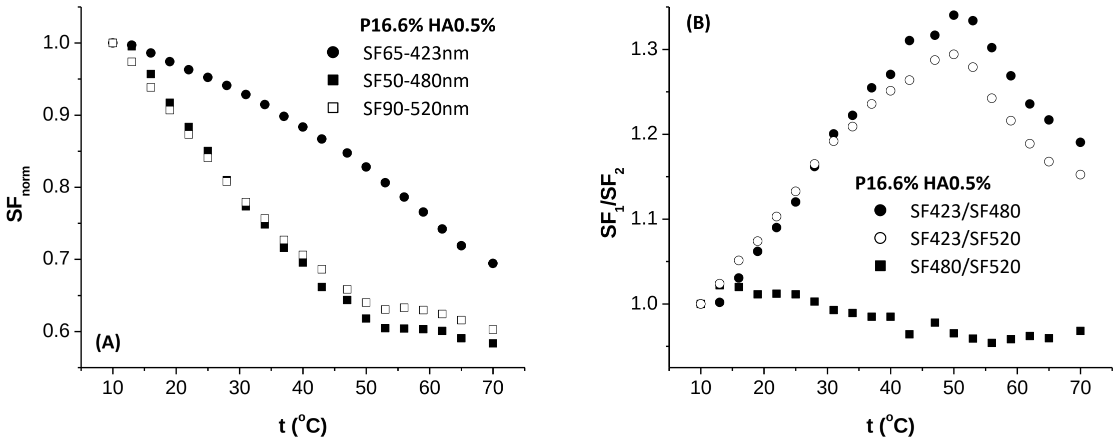

Figure S14. Plots of the synchronous fluorescence intensity (A) and of the ratio of ThT synchronous fluorescence intensities (B) of ThT in P16.6%HA0.5% as a function of temperature.

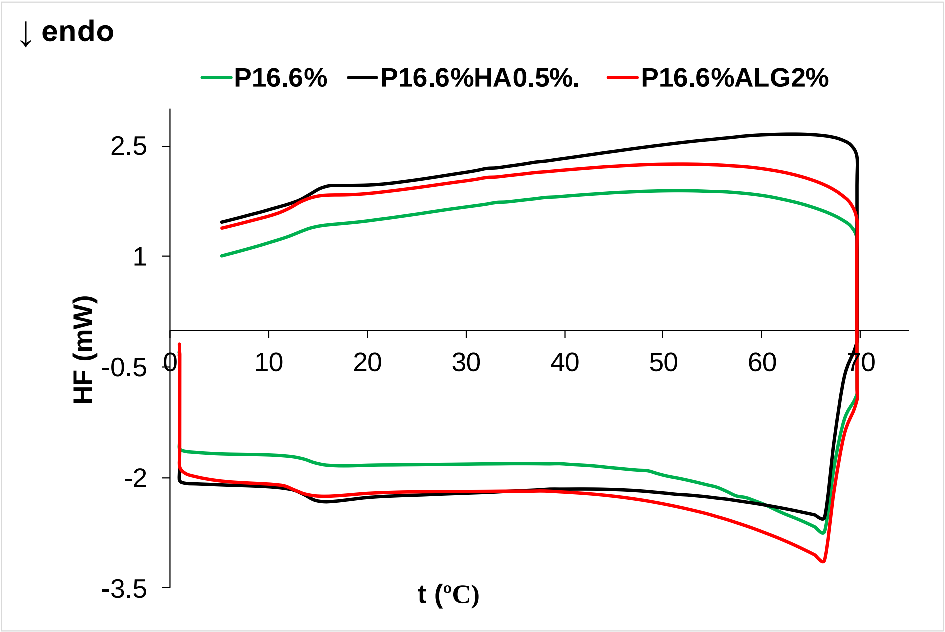

Figure S15. Heating and cooling µDSC scans of P16.6% in the absence and in the presence of HA0.5% or ALG2%.

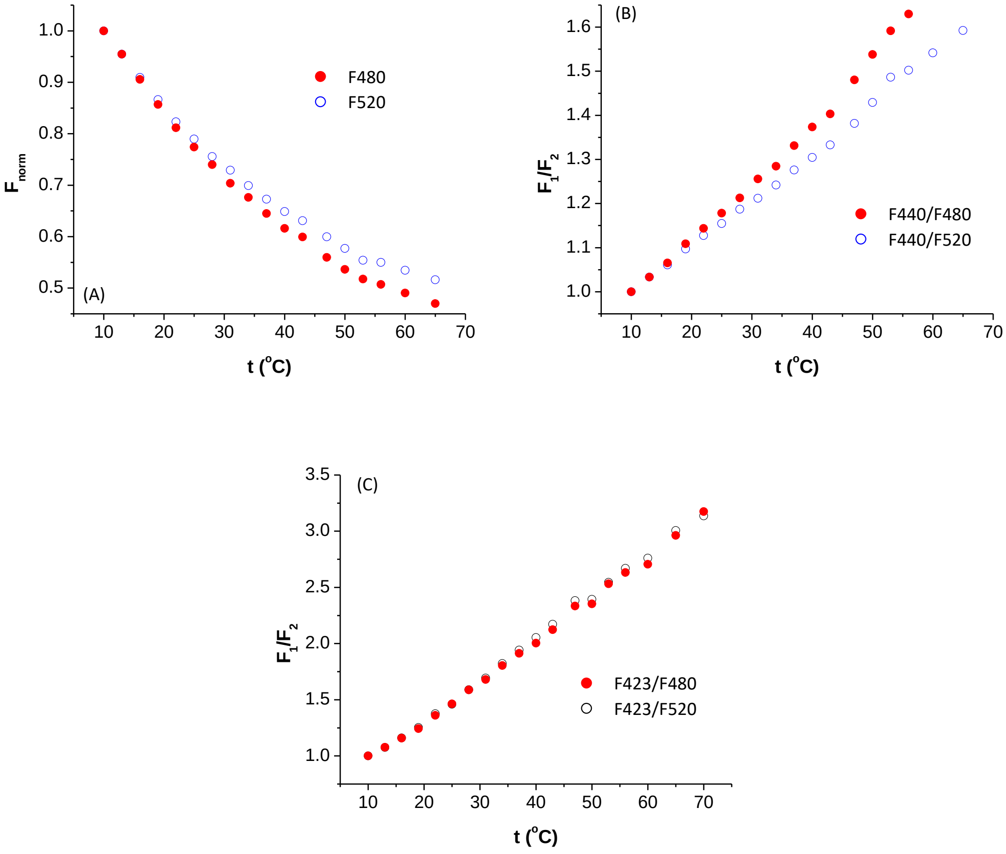

Figure S16. Reference plots for alginate (A – steady-state fluorescence intensities, B – ratio of steady-state fluorescence intensities) and hyaluronic acid (C – ratio of steady-state fluorescence intensities) showing no discontinuity at the gelation temperature.
